# Identifying Cancer Subtypes from miRNA-TF-mRNA Regulatory Networks and Expression Data

**DOI:** 10.1371/journal.pone.0152792

**Published:** 2016-04-01

**Authors:** Taosheng Xu, Thuc Duy Le, Lin Liu, Rujing Wang, Bingyu Sun, Jiuyong Li

**Affiliations:** 1 Institute of Intelligent Machines, Hefei Institutes of Physical Science, Chinese Academy of Sciences, Hefei, Anhui, China; 2 Department of Automation, University of Science and Technology of China, Hefei, Anhui, China; 3 School of Information Technology and Mathematical Sciences, University of South Australia, Adelaide, South Australia, Australia; National Institute of Technology, Rourkela, INDIA

## Abstract

**Background:**

Identifying cancer subtypes is an important component of the personalised medicine framework. An increasing number of computational methods have been developed to identify cancer subtypes. However, existing methods rarely use information from gene regulatory networks to facilitate the subtype identification. It is widely accepted that gene regulatory networks play crucial roles in understanding the mechanisms of diseases. Different cancer subtypes are likely caused by different regulatory mechanisms. Therefore, there are great opportunities for developing methods that can utilise network information in identifying cancer subtypes.

**Results:**

In this paper, we propose a method, weighted similarity network fusion (WSNF), to utilise the information in the complex miRNA-TF-mRNA regulatory network in identifying cancer subtypes. We firstly build the regulatory network where the nodes represent the features, i.e. the microRNAs (miRNAs), transcription factors (TFs) and messenger RNAs (mRNAs) and the edges indicate the interactions between the features. The interactions are retrieved from various interatomic databases. We then use the network information and the expression data of the miRNAs, TFs and mRNAs to calculate the weight of the features, representing the level of importance of the features. The feature weight is then integrated into a network fusion approach to cluster the samples (patients) and thus to identify cancer subtypes. We applied our method to the TCGA breast invasive carcinoma (BRCA) and glioblastoma multiforme (GBM) datasets. The experimental results show that WSNF performs better than the other commonly used computational methods, and the information from miRNA-TF-mRNA regulatory network contributes to the performance improvement. The WSNF method successfully identified five breast cancer subtypes and three GBM subtypes which show significantly different survival patterns. We observed that the expression patterns of the features in some miRNA-TF-mRNA sub-networks vary across different identified subtypes. In addition, pathway enrichment analyses show that the top pathways involving the most differentially expressed genes in each of the identified subtypes are different. The results would provide valuable information for understanding the mechanisms characterising different cancer subtypes and assist the design of treatment therapies. All datasets and the R scripts to reproduce the results are available online at the website: http://nugget.unisa.edu.au/Thuc/cancersubtypes/.

## Introduction

Rather than being a single disease, cancer involves different subtypes characterised by different sets of molecules [1, 2]. Identifying cancer subtypes is a crucial task for selecting the right treatment for patients, as different cancer subtypes may respond well to different treatment therapies. For example, estrogen receptor (ER) positive breast cancer subtype would respond to hormone therapy, and the human epidermal growth factor receptor 2 (HER2) positive subtype is likely to benefit from chemotherapy. However, our current understanding of the mechanisms controlling each cancer subtype is still far from complete.

Several computational methods have been developed to identify cancer subtypes. These methods fall into three different streams of research. In the first stream, data mining or machine learning models are built to utilise gene expression datasets for clustering samples (patients) into different groups, each corresponding to a cancer subtype [3–7]. However, utilising one genomic data type may not be sufficient to identify cancer subtypes accurately. With the advance of sequencing technologies, multiple data types of cancer patients such as genomic, miRNA and related clinical data are made available nowadays. These wealth of datasets lead to the second stream of research in which researchers analyse different types of data separately for identifying subtypes and the results obtained separately are then integrated to form the final result. Highlights of this approach are [1, 8–10]. However, analysing the different types of data separately may lose the complementary information in the data of the same patients, and there may be conflict in the results obtained using different types of data. The last stream of research focuses on analysing multi-omics data at the same time and has identified some important cancer subtypes recently [11–14].

However, the information from gene regulatory networks is rarely used by the existing computational methods. Gene regulatory networks play an important role in every life process, and understanding the dynamics of these networks help reveal the mechanisms of diseases [15]. Although the importance of network-based information has been addressed in recent works [16, 17], there is still a lack of methods utilising biological information from networks to identify cancer subtypes. Moreover, it remains a great challenge to associate the multi-omics data and network information with cancer subtypes and the outcomes in particular prognosis. Recently, Liu et al. [18] proposed the NCIS (network-assisted co-clustering for the identification of cancer subtypes) method to utilise the expression profiles of mRNAs and the network information of mRNA-mRNA interactions with a bi-clustering method to discover cancer subtypes. However, gene regulatory networks are complex and involve many types of regulators including miRNAs and TFs. It is of interest to utilise the information in the networks that involve miRNAs, TFs, and mRNAs in identifying cancer subtypes. The information may not only improve the accuracy of the computational models, but also provide insights into the mechanisms (the regulatory networks) regulating each cancer subtype.

In this paper, we propose a method, called weighted similarity network fusion (WSNF), to identify cancer subtypes by making use of both the expression data and network information of miRNAs, TFs and mRNAs. Given a dataset containing the expression profiles of a set of miRNAs, TFs and mRNAs (known as features in the rest of the paper), WSNF firstly retrieves the interactions between these features from different interatomic databases to build the miRNA-TF-mRNA regulatory network. In the network, features are represented by nodes and interactions between features are indicated by edges. We then calculate the weight (i.e. importance) of a feature by utilising the miRNA-TF-mRNA network information and the expression variation of the features. Finally, we modify the similarity network fusion (SNF) approach [11] to take the feature weight into consideration when clustering patients for identifying cancer subtypes.

We apply the WSNF method to the TCGA breast cancer and GBM datasets. The experimental results show that our method has successfully identified five breast cancer subtypes and three GBM subtypes which show significantly different survival patterns. The information from the miRNA-TF-mRNA regulatory network improves the performance of the network fusion approach, as the WSNF method performs better than both SNF [11], the network fusion method without using feature weight and NCIS [18] that uses only mRNA expression data and mRNA-mRNA interactions. We also compare our method with Consensus clustering (CC) [7], a method that is commonly used in TCGA research. The experimental results show that the WSNF method also has better performance with both the breast cancer and GBM datasets. For the breast cancer dataset, we analyse the identified subtypes in detail and report the results in terms of the expression patterns, the differences in the miRNA-TF-mRNA regulatory networks across the different subtypes, and the functional pathways characterising each subtype. The information can be valuable for assisting the treatment design of specific breast cancer subtypes.

## Materials and Methods

### Method overview

**Fig 1:**
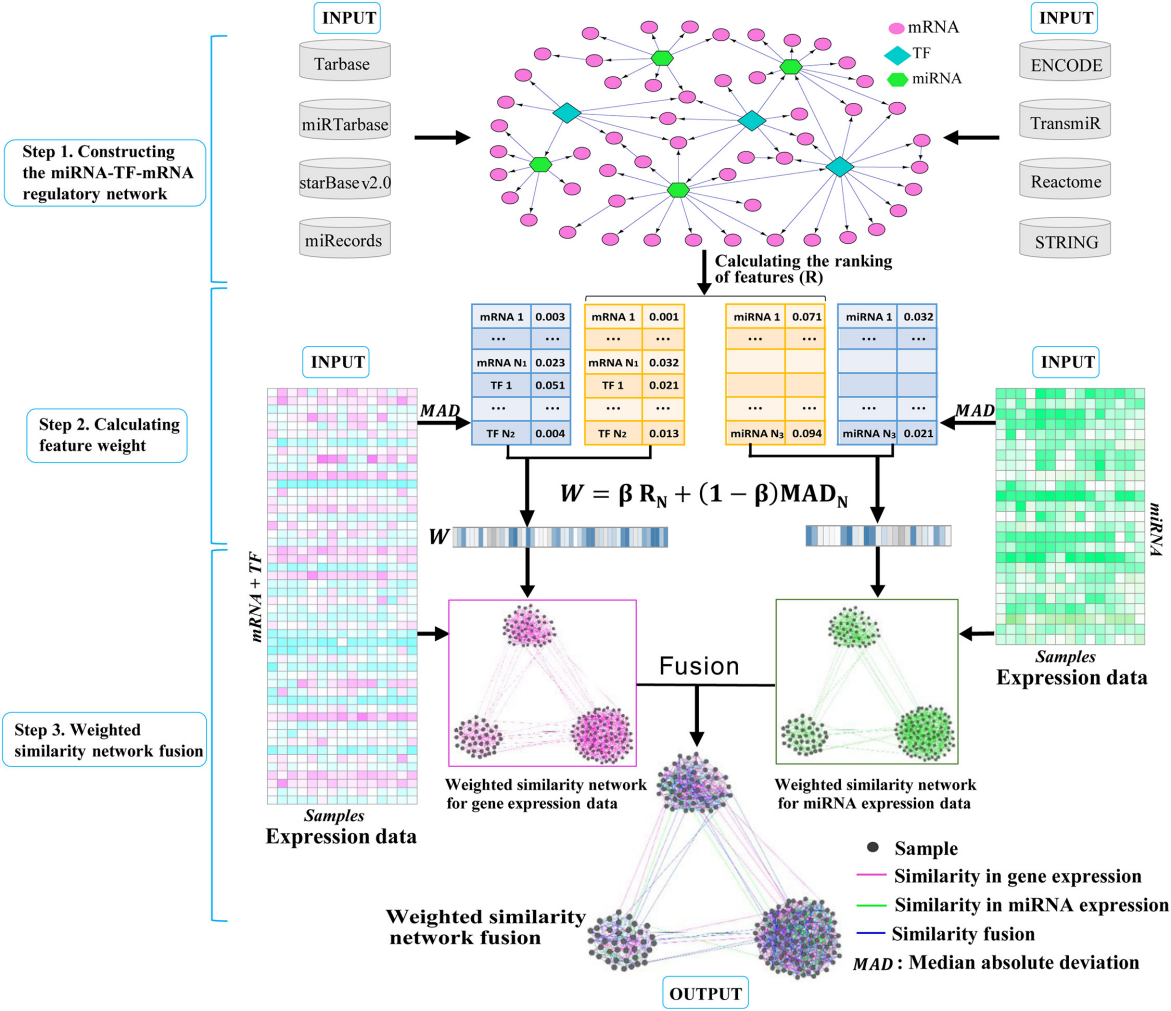
Workflow of WSNF. In step 1, interactions between miRNAs, TFs and mRNAs obtained from the databases are used to construct the miRNA-TF-mRNA regulatory network. In step 2, the ranking of each feature (R) is calculated based on the network information, and gene and miRNA expression data are used to get the feature expression variation (MAD) across all the samples. Then for each feature, its ranking and expression variation are combined to obtain its weight (W). In step 3, the weighted sample similarity networks are obtained from genes (mRNAs, TFs) and miRNAs separately using the weights and expression data of the features, and finally network fusion and clustering are performed to find patient groups that imply cancer subtypes.

We propose to use the miRNA-TF-mRNA regulatory network to assist the identification of cancer subtypes. There are three main steps in the WSNF method (Fig 1), including: 1) constructing miRNA-TF-mRNA regulatory network, 2) calculating the weight for each feature (miRNA, TF, mRNA), and 3) modifying and applying the similarity network fusion approach [11] to identify cancer subtypes, while taking the feature weight into consideration. We describe the details of each step in the following.

### Constructing the miRNA-TF-mRNA regulatory network

**Fig 2:**
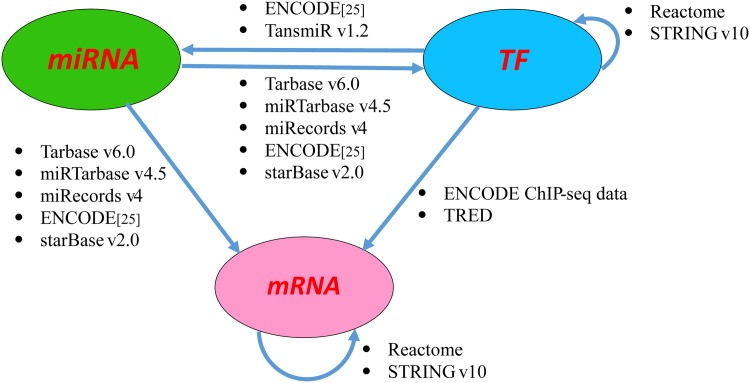
The data sources for constructing the miRNA-TF-mRNA regulatory network.

In this step, we use a variety of sources to build the miRNA-TF-mRNA interaction networks. The network contains different types of interactions, including those between miRNA-mRNA, miRNA-TF, TF-miRNA, TF-mRNA, TF-TF, and mRNA-mRNA. Fig 2 shows the details of the data sources for retrieving the different type interactions. In the figure, each type of the interactions is represented as a link where the source is the regulator and the arrow end is the target. The data sources are listed next to each type of the interactions.

We firstly get the list of TFs by combining the TFs in the Encyclopedia of DNA Elements (ENCODE) ChIP-seq data, TransmiR [19] and FANTOM5 Human transcription factors which are available at http://fantom.gsc.riken.jp/5/sstar/Browse_Transcription_Factors_hg19. Finally a list of 1679 TFs is obtained (see the S1 File for the list).

As shown in Fig 2, we obtain the miRNA-mRNA and miRNA-TF interactions from experimentally confirmed databases, including Tarbase [20], mirTarbase [21], mirRecords [22], and prediction database starBase v2.0 [23]. Tarbase, mirTarbase and mirRecords include the curated confirmed interactions from the literature. starBase v2.0 contains the union of the sets of miRNA-mRNA interactions predicted by the five miRNA target prediction software programs (TargetScan, PicTar, PITA, miRanda and RNA22). It also tests each of the miRNA-mRNA interaction pairs based on TCGA Pan-cancer [24] expression datasets. The criterion of the validation test is the anti-correlation with negative Pearson correlation coefficient (*p*-value< 0.05) between a miRNA and its target. In our network, we use the miRNA-mRNA interactions in starBase v2.0 that are supported by at least one TCGA Pan-cancer expression dataset. In addition, the miRNA-mRNA interactions derived from ENCODE data [25] are also used in our work. The interactions are available at: http://encodenets.gersteinlab.org/.

The mRNA-mRNA interactions are retrieved from Reactome [26] and STRING v10.0 [27]. Since contained in the Reactome and STRING are the protein-protein interaction pairs, we use the *org.Hs.eg.db* R package [28] to map the protein-gene annotation to get the corresponding mRNA-mRNA interaction pairs. We choose the score cut-off as 0.9 in STRING v10.0 to select the mRNA-mRNA pairs of high credibility for our network.

For TF regulation, we obtain the interactions between TF-mRNA from the ENCODE ChIP-seq data [29] and Transcriptional Regulatory Element Database (TRED) [30]. ENCODE ChIP-seq data at UCSC Genome Browser are processed using the computational pipeline to generate uniform peaks of TF binding. TRED is an integrated repository for both cis- and trans-regulatory elements. It contains the curated transcriptional regulation information, including the transcription factor binding motifs and experimental evidence. We retrieve the TF-TF interactions from Reactome and STRING, with the protein-gene annotation mapping as that for getting the TF-TF interactions. For our network, TF-miRNA interactions are obtained from two sources: TransmiR [19] and the supplementary data of [25] that is also available at http://encodenets.gersteinlab.org/.

### Calculating feature weights

With the proposed WSNF method, we calculate the weight of a feature in two stages. Firstly, we use the information of the miRNA-TF-mRNA network constructed in the previous step to rank the features. Then the expression data is used to find the expression variation of each feature across all the samples in the datasets. At last, the weight of a feature is obtained by combining its ranking and expression variation.

#### Stage 1: Computing ranking of features using Google PageRank

Google PageRank [31, 32] is an algorithm which was initially used to rank the vast number of webpages by Google Search. It is based on a directed graph *G*(*V*,*E*) where the nodes *V* represent webpages and the edges *E* indicate the hyperlinks between the webpages. The basic assumption is that an important webpage is likely to have more inbound links from other webpages. Suppose there are *N* webpages {*p*_1_, *p*_2_, …, *p*_*N*_}. The ranking of a webpage *p*_*i*_ is defined as the following:

$$PR\left(p_{i}\right)=\frac{1-d}{N}+d\sum_{p_{j}\in M\left(p_{i}\right)}\frac{PR(p_{j})}{L(p_{j})}$$ (1)

where *PR*(*p*_*i*_) and *PR*(*p*_*j*_) are the rankings of webpages *p*_*i*_ and *p*_*j*_ respectively, with *p*_*i*_ ← *p*_*j*_ ; *d* is the damping factor which is like a click-through probability used to decay the ranking of the webpages with no outgoing links, and 0 < *d* < 1; *M*(*p*_*i*_) is the set of webpages that are linked to *p*_*i*_ ; and *L*(*p*_*j*_) is the number of outbound links from *p*_*j*_. So a webpage *p*_*i*_ will have a high ranking if it is linked by many other high-ranked webpages *p*_*j*_. For interested readers, the convergence and computation of the PageRank using the above iterative formula (i.e. Eq 1) are illustrated in [33, 34].

For our case of utilising miRNA-TF-mRNA regulatory network to rank a feature, a molecular regulating many targets is important. In our miRNA-TF-mRNA network, denoted as *G*(*V*,*E*), the nodes *V* are the features (miRNAs, TFs and mRNAs) and the edges *E* are the interactions between regulators and their targets. The direction of an edge is from a regulator to its target. An important regulator is analogous to an important webpage in PageRank that many other webpages link to, except that the regulator has many links going out of it to its targets. Suppose there are *N* features {*f*_1_, *f*_2_, …, *f*_*N*_}. The ranking (regulatory importance) of a feature *f*_*i*_ can be defined as follows using a modified PageRank algorithm:

$$R\left(f_{i}\right)=\frac{1-d}{N}+d\sum_{f_{j}\in T\left(f_{i}\right)}\frac{R(f_{j})}{L(f_{j})}$$ (2)

where *R*(*f*_*i*_) and *R*(*f*_*j*_) are the rankings of features *f*_*i*_ and *f*_*j*_ respectively, with *f*_*i*_ → *f*_*j*_ ; *d* is the the damping factor, and 0 < *d* < 1; *T*(*f*_*i*_) is the set of targets that *f*_*i*_ regulates; and *L*(*f*_*j*_) is the number of regulators which regulate *f*_*j*_.

The R and Matlab scripts of computing the feature ranking from miRNA-TF-mRNA regulatory network is provided in the S2 File.

#### Stage 2: Integrating feature ranking and feature variation

The expression variation across samples is an important indicator for the research of cancer genomic data. The features (e.g. genes) with higher expression variations are always treated as more important biological marker in cancer mechanisms. We use the median absolute deviation (MAD) to represent the expression variation of a feature. The MAD of a feature *f*_*i*_ is calculated as:

$$MAD\left(f_{i}\right)=median(|X\left(f_{i}\right)-median\left(X\left(f_{i}\right)\right)\left|\right)$$ (3)

where *X*(*f*_*i*_) is a numeric vector which represents the expression values of feature *f*_*i*_ across all samples (patients).

To integrate the feature variation with feature ranking, NCIS [18] follows the idea of GeneRank [35] to simply replace the part [$\frac{1-d}{N}$] in Google PageRank algorithm with the MAD to obtain the final weight of a feature. However, we find that the final weight obtained in this way by both GeneRank and NCIS is strongly correlated with the feature weight directly calculated with Eq 2, i.e. without using MAD. The strong correlation implies that the approach taken by the two methods of integrating MAD is not effective as the expression variation information is not reflected by the final weight obtained using their approach. The detailed results on this finding are shown in the S3 File.

To overcome this problem, we adopt a linear model to effectively integrate the feature ranking and the feature variation in this paper. We firstly normalise the feature ranking obtained from the miRNA-TF-mRNA regulatory network and feature variation from expression data as follows:

$$R_{N}\left(f_{i}\right)=\frac{R(f_{i})}{\Sigma_{m=1}^{N}R\left(f_{m}\right)}$$ (4)

$$MAD_{N}\left(f_{i}\right)=\frac{MAD(f_{i})}{\Sigma_{m=1}^{N}MAD\left(f_{m}\right)}$$ (5)

A linear model is then applied to integrate these two measures to get the final weight for each feature.

$$W\left(f_{i}\right)=\beta R_{N}\left(f_{i}\right)+\left(1-\beta \right)MAD_{N}\left(f_{i}\right)$$ (6)

where *β* is a tuning parameter for the importance of the miRNA-TF-mRNA regulatory network information. The larger the value of *β* is the more important role the information of the miRNA-TF-mRNA regulatory network will play in calculating the final weight of the features. In our experiments, we set *β* to 0.8 to focus more on the network information for the cancer subtype discovery.

### Weighted similarity network fusion

We utilise the feature weight information to assist the identification of cancer subtypes from the gene expression data and miRNA expression data. To this end, we modify the similarity network fusion (SNF) method [11] to incorporate the feature weight obtained in the previous step into the process of cancer subtype classification.

SNF is a multi-omics data processing method that constructs a fusion patient similarity network by integrating the patient similarity obtained from each of the genomic data types. SNF calculates the similarity between patients using each single data type separately. The similarities between patients from different data types are then integrated by a cross-network diffusion process to construct the fusion patient similarity matrix. Finally, a clustering method is applied to the fusion patient similarity matrix to cluster patients into different groups, which imply different cancer subtypes.

The key step of SNF is to define the similarity between patients, as we need to stratify similar patients into the same group (subtype). Euclidean distance is used in SNF to measure the similarity between patients in single genomic data type, where, however, all features are treated as equally important. Suppose that there is an expression profile dataset (*n* patients × *p* features), then the Euclidean distance between patient *S*_*i*_ and patient *S*_*j*_ is:

$$Distance\left(S_{i},S_{j}\right)=\sqrt{\sum_{m=1}^{p}{\left(f_{m}^{S_{i}}-f_{m}^{S_{j}}\right)}^{2}};\forall i,j\leq n,i\neq j$$ (7)

where $f_{m}^{S_{i}}$ and $f_{m}^{S_{j}}$ are the expression values of *f*_*m*_ in patients *S*_*i*_ and *S*_*j*_, respectively.

We modify the patient distance formula as follows take the weight of each feature into consideration:

$$Distance\left(S_{i},S_{j}\right)=\sqrt{\sum_{m=1}^{p}W\left(f_{m}\right)*{\left(f_{m}^{S_{i}}-f_{m}^{S_{j}}\right)}^{2}};\forall i,j\leq n,i\neq j$$ (8)

By using the above modified samples distance formula, the proposed WSNF method considers similarity of two patients based on not only the overall difference between the expression levels of all their features, but also the importance (weight) of each of the features. As we make use of the miRNA-TF-mRNA network information in the calculation of feature weight and our method treats different features differently, we will see in the Results and discussion Section that WSNF significantly outperforms the SNF and the other commonly used methods for identifying cancer subtypes.

## Results and Discussion

### Datasets

In this paper, we use the BRCA and GBM datasets from The Cancer Genome Atlas (TCGA) for our experiments, including the gene (mRNA and TF) expression data, miRNA expression data and clinical data (overall survival time, survival status and some clinical covariates). The Level 3 TCGA tumor samples are downloaded from the Broad GDAC Firehose (timestamp: 2015-04-02). To get the most number of matched samples for both cancers, we use RNASeq and miRNAHiseq data for BRCA and microarray data for GBM.

The genes and miRNAs with very low expression levels and low variations across samples are removed. The different cut-off points are selected based on the distribution characteristics of the BRCA and GBM datasets (see the S3 File). For the BRCA RNASeq and miRNAHiseq datasets, we firstly use the *log*2 transformation to preprocess them, which is commonly used for RNA-sequencing data as introduced in the *DESeq2* [36] R package. We calculate the average value for each feature across samples and remove the 25% genes and 60% miRNAs with low average expression. Then the standard deviation of each gene and miRNA is calculated, and genes and miRNAs with standard deviation less than 0.5 are also removed. For the GBM microarray data, there are some missing observations. We firstly apply the imputation by using the *impute* R pacakage [37]. Then we calculate the standard deviation of each gene and miRNA. The genes with standard deviation less than 0.6 and the miRNAs with standard deviation less than 0.2 are removed. The detailed processing procedure of the datasets are recorded in the S3 File. In the end, there are 587 matched samples in BRCA with 12,233 mRNAs, 1,338 TFs and 361 miRNAs. Meanwhile, for GBM there are 276 matched samples with 10,278 mRNAs, 1,083 TFs and 287 miRNAs (see the S3 File).

### Network construction

As mentioned in the Materials and Methods Section, we use several public databases to construct the miRNA-TF-mRNA regulatory network. Table 1 shows the number of interactions from the data sources for constructing the regulatory networks for the BRCA dataset. Similar information for the GBM dataset is in the S3 File.

**Table 1:** The interactions used for constructing the miRNA-TF-mRNA regulatory network for the BRCA dataset.

	Database	Total interactions	Found interactions
	Tarbase v6.0	17,526	12,130
miRNA → TF&	miRTarBase v4.5	37,423	26,847
miRNA →mRNA	miRecords v4	1,707	1,095
	starBase v2.0	320,709	219,088
	ENCODE [25]	117,193	54,603
TF → miRNA	ENCODE [25]	1,648	579
	Transmir v1.2	649	457
TF → mRNA	ENCODE ChIP-Seq	229,486	133,952
	TRED	7,066	4,739
TF → TF&	Reactome	127,452	60,648
mRNA → mRNA	STRING v10.0	250,843	122,938

### The identified subtypes have significantly different survival patterns

**Fig 3:**
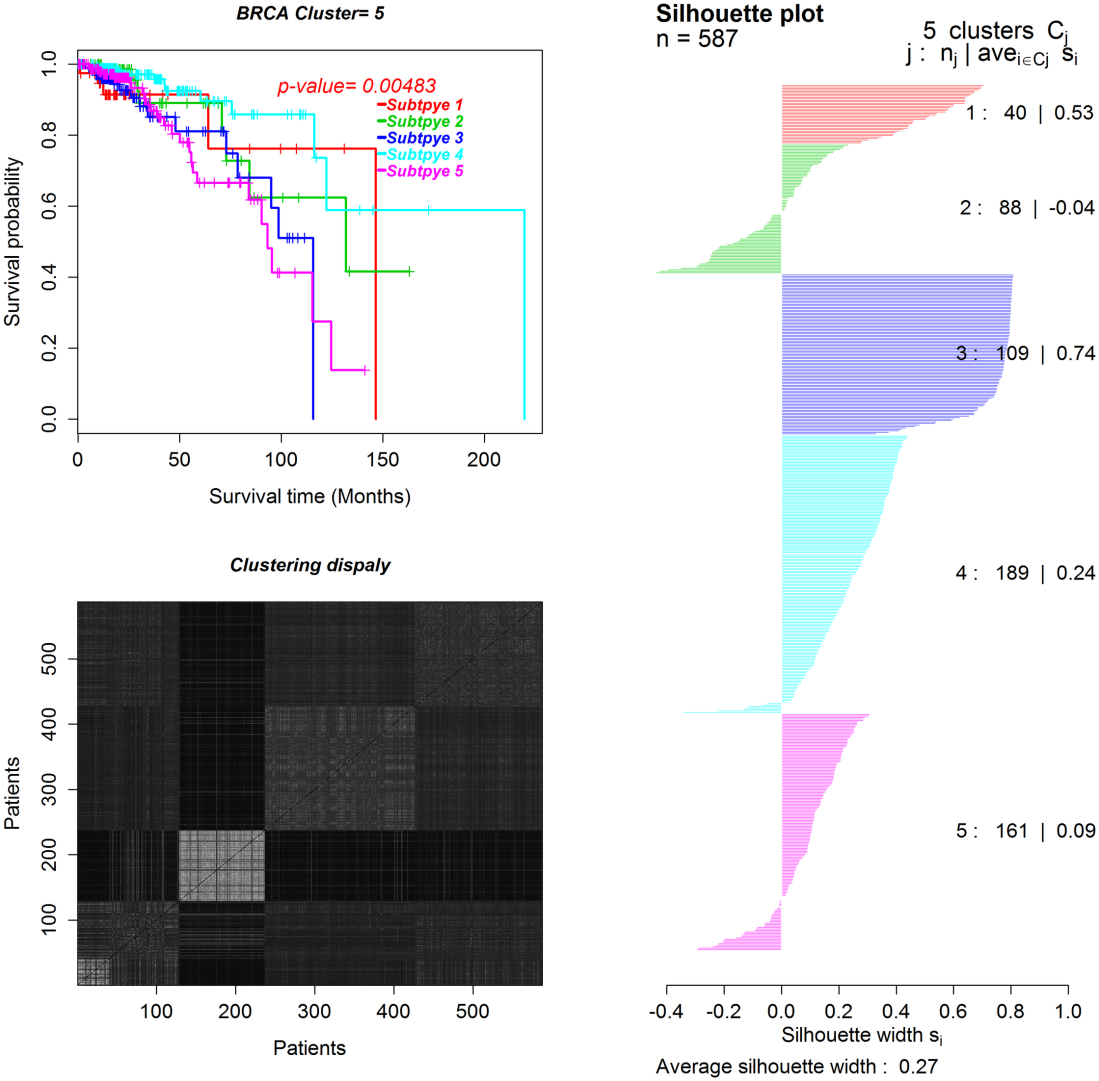
The survival curves and Silhouette plots for the five subtypes of BRCA. *j*, *n*_*j*_, *s*_*i*_ in the Silhouette plot are subtype label, the number of patients in the subtype and the Silhouette width for patient *i*, respectively.

**Fig 4:**
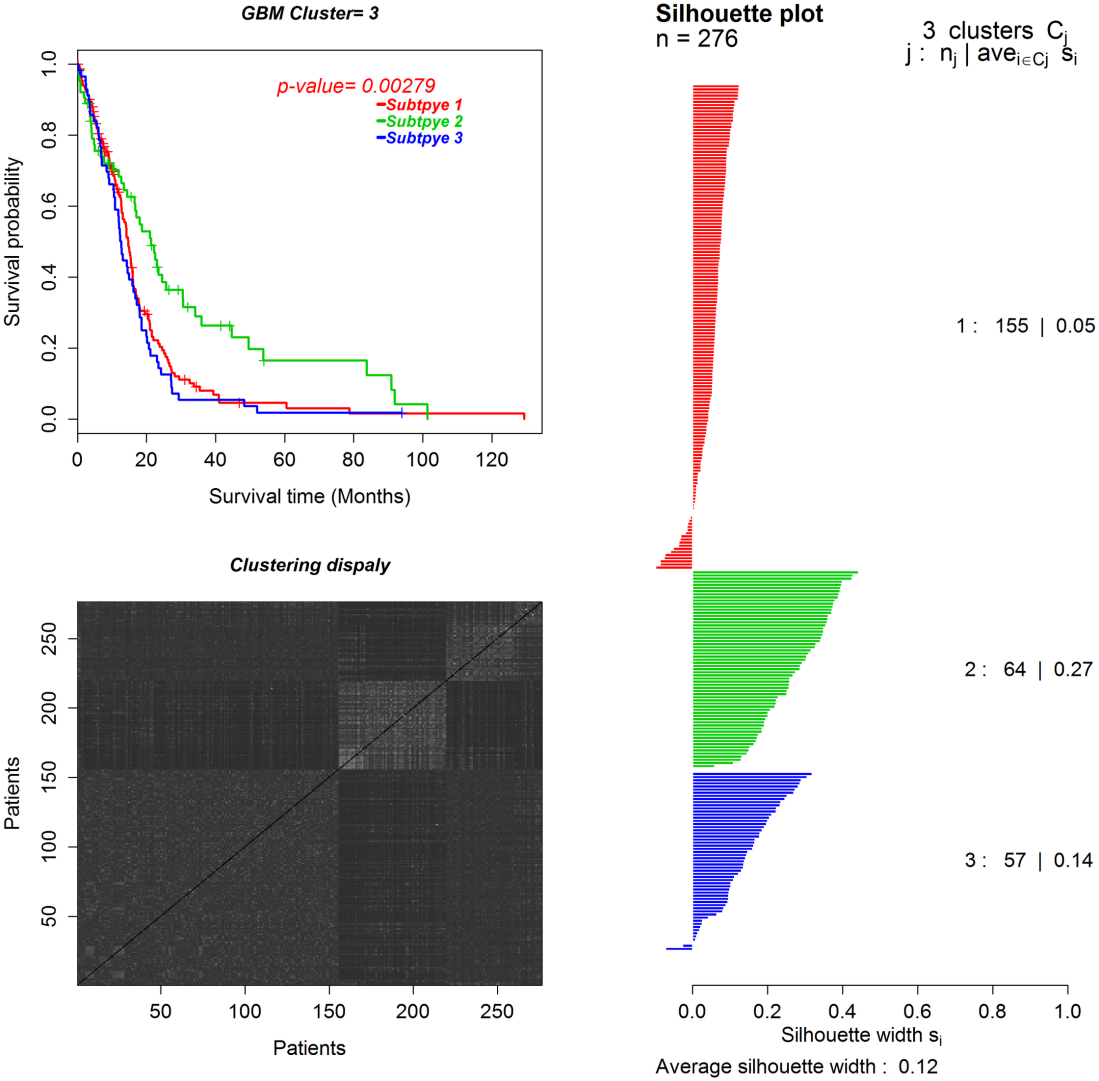
The survival curves and Silhouette plots for the three subtypes of GBM. *j*, *n*_*j*_, *s*_*i*_ in the Silhouette plot are subtype label, the number of patients in the subtype and the Silhouette width for patient *i*, respectively.

With the constructed networks and the BRCA and GBM expression datasets, WSNF identifies five breast cancer subtypes and three GBM subtypes. The identified cancer subtypes and related clinical information for breast cancer and GBM are given in the S4 and S5 Files. To assess how well our method has performed in identifying cancer subtypes, we conduct survival analysis of the identified cancer subtypes. Figs 3 and 4 show the survival curves of the patients in the five subtypes of BRCA and the three subtypes of GBM, respectively. The *p*-values from the Log-rank tests [38] are 0.00483 for BRCA and 0.00279 for GBM. The p-values suggest that the identified subtypes in both datasets have significantly different survival patterns, indicating different cancer subtypes respectively.

Furthermore, we use the Silhouette width [39] and black-white heatmap to demonstrate the consistency of the samples (patients) in each subtype and the difference across different subtypes, respectively. As shown in Figs 3 and 4, the overall average Silhouette width values are positive for both BRCA and GBM. Note that the Silhouette width value is positive if the samples in each subtype are consistent, and negative otherwise. Meanwhile, the black-white heatmaps are generated from the matrix of sample similarity by arranging the samples according the cluster labels. The block boundaries for all subtypes are very clear. In particular, the third subtype of BRCA has a high Silhouette width value and a clear contrast in the black-white heatmap, which suggests unique characteristics of the patients in this subtype.

### The network information improves the identification of cancer subtypes

To investigate whether the information from the miRNA-TF-mRNA regulatory network actually helps improve the identification of cancer subtypes, we compare the WSNF method with the previously proposed methods including NCIS [18], Consensus clustering (CC) [7], and SNF [11]. NCIS utilises gene expression data and the information from mRNA-mRNA interactions. CC is the commonly used clustering method in TCGA research papers [1, 8, 40–42] based on single genomic data type. SNF is the multiple genome data fusion and clustering method but does not use the information from the gene regulatory networks. To make a fair comparison, from our processed datasets (BRCA & GBM) and constructed miRNA-TF-mRNA regulatory networks, we use the gene expression data and extract mRNA-mRNA interactions as the input for NICS. We concatenate the normalised gene expression data and normalised miRNA expression data for each patient as the input data for CC. The inputs of the SNF are the gene expression data and miRNA expression data. The inputs of our WSNF method are the gene expression data, miRNA expression data and the miRNA-TF-mRNA regulatory networks. We conduct the survival analyses for the identified subtypes by each of the methods and compare the *p*-values of the Log-rank tests [38] to evaluate the significance of the different survival distributions across subtypes.

From Table 2, we see that WSNF has significantly lower *p*-values than other common methods in both the BRCA and GBM datasets. When *β* is set to 1, the weight for the features is completely determined by the miRNA-TF-mRNA regulatory network. The results show that the WSNF method is better than the other existing methods, suggesting that the information from the miRNA-TF-mRNA regulatory network helps improve the identification of the subtypes. We observe further that the method performs very well in both datasets when *β* is 0.8 (which is default value used for *β*).

**Table 2:** Comparison of the Log-rank tests of cancer subtypes identified by different methods.

Dataset	NCIS	CC	SNF	WSNF(*β* = 1)	WSNF(*β* = 0.8)
BRCA	0.374	0.0634	0.0583	0.0277	**0.00483**
GBM	0.091	0.321	0.0107	0.00364	**0.00279**

### Breast cancer subtypes show different expression patterns

**Fig 5:**
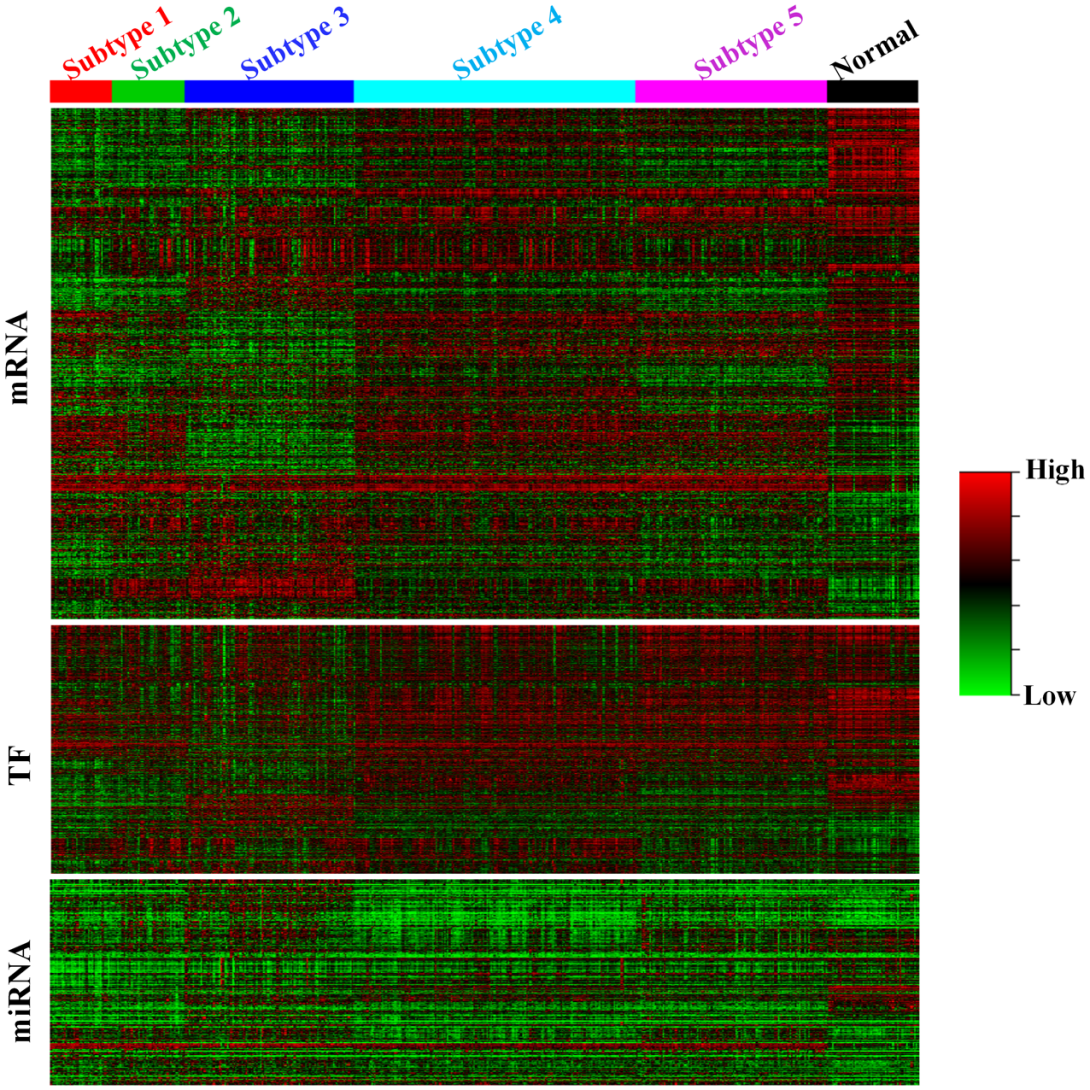
mRNA, TF and miRNA expression heatmap for BRCA dataset.

In the previous section, we have demonstrated the performance of WSNF using the BRCA and GBM datasets. The results suggest that WSNF is capable of discovering cancer subtypes with distinct survival patterns and our method outperforms the existing cancer subtype identification methods. We investigate the mRNA, TF and miRNA expression patterns across the five different breast cancer subtypes. Similar to [8], we extract the “core samples” which are identified on the basis of their Silhouette width by removing samples with negative Silhouette width values in each subtype. There are 502 samples with positive Silhouette width values across the five subtypes. We also obtain 69 normal samples from TCGA for comparison. The heatmaps for mRNA, TF, and miRNA expression are shown in Fig 5. Taking normal group as the reference, we can see from the figure that the expression profiles between the subtypes are significantly different.

**Fig 6:**
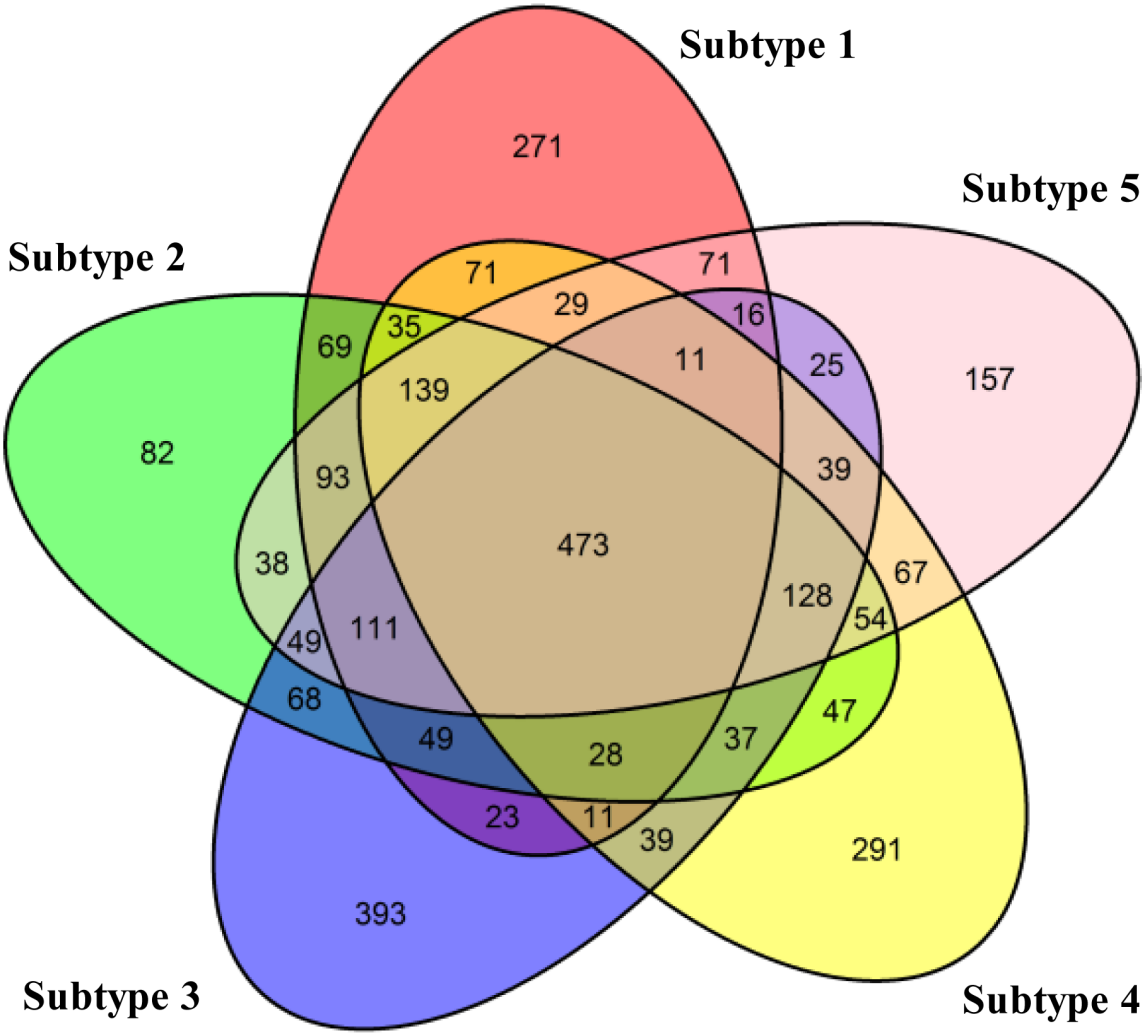
The overlap of the differentially expressed genes across the five subtypes of BRCA.

**Fig 7:**
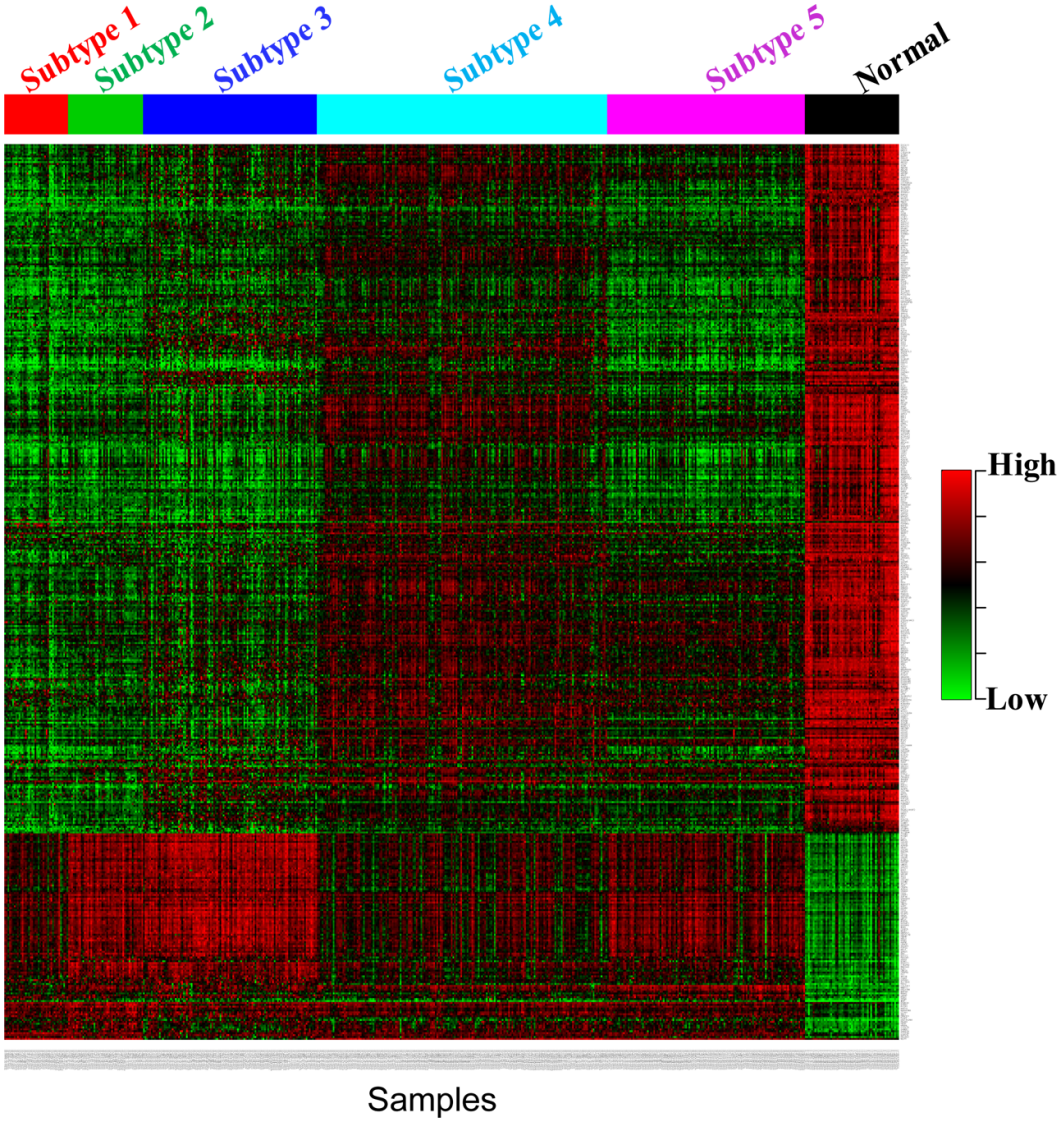
The heatmap of 473 common differentially expressed genes in the five subtypes of BRCA.

To have a closer look at the expression patterns of genes characterising each subtype, we use the *Voom* [43] method and *Limma* [44] R Package to find the differentially expressed genes (adjusted *p*-value<0.01) between each subtype and normal samples. We select the top 1500 differentially expressed genes in each subtype for the analysis. Fig 6 shows the overlap of differentially expressed genes across the subtypes. There are 473 common differentially expressed genes for all subtypes. Meanwhile, each subtype has their specific genes (Subtype 1: 271, Subtype 2: 82, Subtype 3: 393, Subtype 4: 291, Subtype 5: 157). The common genes across the five subtypes and the subtype-specific genes are listed in the S6 File. Although there are some common differentially expressed genes for all subtypes, their expression patterns are quite different as shown in Fig 7. In the latter section, we conduct the pathway analysis for the subtype-specific genes to explore their function characteristics in each subtype.

### Alterations in regulatory networks across breast cancer subtypes

**Fig 8:**
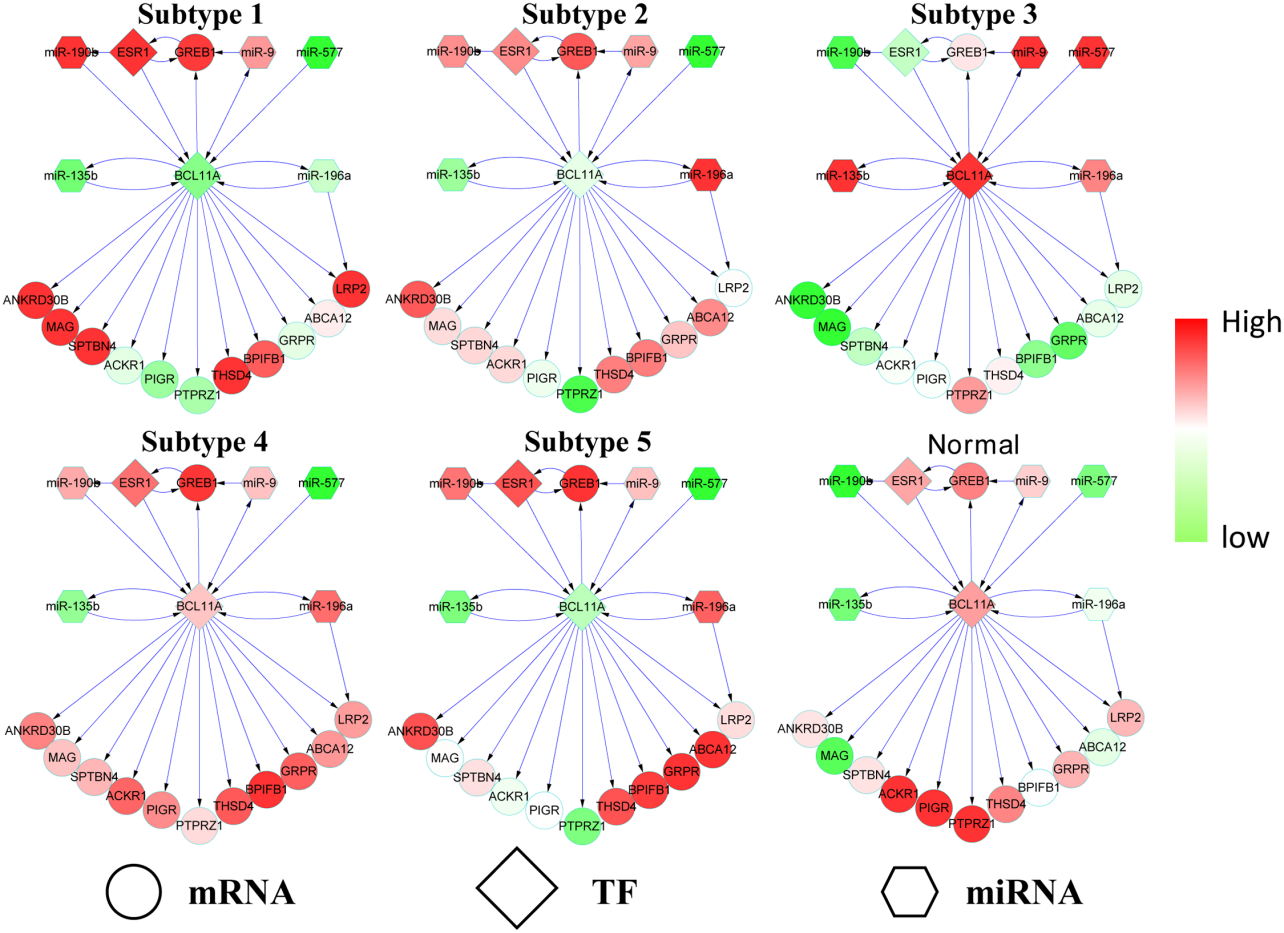
The expression patterns of miRNAs, TFs and mRNAs in the BCL11A network.

We extract the TF gene *BCL11A* to show the alterations in the miRNA-TF-mRNA regulatory network across the identified breast cancer subtypes. *BCL11A* is a proto-oncogene that has a significant effect on breast cancer [45]. As shown in Fig 8, *BCL11A* is highly expressed in Subtype 3, but lowly expressed in other subtypes. We map the patients in Subtype 3 to clinical data and find that 73.5% of the patients are in triple-negative class, including ER-, PR- and HER2-. This is consistent with the results in [45], which proved that *BCL11A* is highly expressed in triple-negative breast cancer.

The target genes of *BCL11A*, including *ANKRPD30B, MAG, BPIFB1* and *GRPR* are lowly expressed in Subtype 3 and this pattern is opposite to those in other subtypes. These different patterns suggest that *BCL11A* down regulates *ANKRPD30B, MAG, BPIFB1* and *GRPR* in breast cancer, and the expression level of *BCL11A* may be a marker of different subtypes. We also observe the co-expression between *BCL11A* and *PTPRZ1* in all subtypes and normal samples. However, the expression level of *BCL11A* and *PTPRZ1* are different across different subtypes and normal samples, suggesting that the co-expression of *BLC11A* and *PTPRZ1* are specific to the subtypes.

To investigate why *BCL11A* has low expression levels in Subtypes 1, 2 and 5 (and not very high in Subtype 4), we observe the changes in the expression levels of its upstream regulators. As in Fig 8, *miR-190b* and *ESR1* have high expression levels in the three subtypes, which is totally opposite to that in Subtype 3. This observation suggests that *miR-190b* and *ESR1* may down regulate *BCL11A* in breast cancer. The level of down regulation may characterise different breast cancer subtypes. We believe that the information of miRNA-TF-mRNA regulatory mechanisms across subtypes would provide insights into the cause of each subtype.

### Top enriched pathways in different breast cancer subtypes

To investigate the pathways involved in each subtype, we conduct the pathway analysis on the differentially expressed genes characterising each subtype as shown in Fig 6. We use GeneGO Metacore^*TM*^ (https://portal.genego.com/) to select the top 5 significant pathways for each subtype. The genes to be analysed are the subtype-specific genes (Subtype 1: 271, Subtype 2: 82, Subtype 3: 393, Subtype 4: 291, Subtype 5: 157), as we wish to observe different biological pathways for different subtypes. We also conduct the pathway analysis for the 473 common genes in all the subtypes.

Table 3 shows the top 5 enriched pathways of the common genes and subtype-specific genes of each subtype. We can see from the table that the pathways are quite different between different subtypes. The significant pathways of the common genes are related to cell cycle. Pathways in Subtype 1 are related to Epithelia to Mesenchymal Transition (EMT), which implies the progression of breast carcinoma to metastasis [46]. Meanwhile, pathways in Subtype 3 are related to the neurophysiological process, Subtype 4 pathways are about the cytoskeleton remodeling, and Subtype 5 pathways are related to the immune responses. These pathways show that different subtypes have different causes.

**Table 3:** Top 5 enriched pathways in five subtypes of BRCA. The *p*-values have been adjusted by the Benjamini-Hochberg (BH) method.

Datasets	Top 5 enriched pathways	Adj- *p* -value
Common	Cell cycle The metaphase checkpoint	1.337E-15
	Cell cycle Role of APC in cell cycle regulation	1.118E-10
	Cell cycle Spindle assembly and chromosome separation	5.812E-08
	Reproduction Progesterone-mediated oocyte maturation	3.553E-07
	Cell cycle Chromosome condensation in prometaphase	3.947E-07
Subtype 1	NETosis in SLE	3.209E-06
	Development WNT signaling pathway.Part 2	7.827E-05
	Cell adhesion Cell-matrix glycoconjugates	1.536E-04
	Hypoxia-induced EMT in cancer and fibrosis	1.969E-04
	Immune response IL-12 signaling pathway	2.404E-04
Subtype 2	Immune response IL-6 signaling pathway	4.784E-03
	Neurophysiological process Receptor-mediated axon growth repulsion	9.892E-03
	Signal transduction IP3 signaling	1.165E-02
	Immune response Function of MEF2 in T lymphocytes	1.258E-02
	Development Role of HDAC and calcium	1.403E-02
Subtype 3	Cell adhesion ECM remodeling	8.725E-04
	Breast cancer (general schema)	2.768E-03
	Neurophysiological process Melatonin signaling	3.299E-03
	Neurophysiological process Receptor-mediated axon growth repulsion	3.896E-03
	Action of GSK3 beta in bipolar disorder	4.203E-03
Subtype 4	Cell adhesion Gap junctions	1.212E-05
	Cytoskeleton remodeling Neurofilaments	1.132E-04
	Cytoskeleton remodeling Keratin filaments	4.832E-04
	Cell adhesion Tight junctions	4.832E-04
	Breast cancer (general schema)	7.987E-04
Subtype 5	Development Prolactin receptor signaling	4.736E-05
	Immune response ETV3 affect on CSF1-promoted macrophage differentiation	7.416E-05
	Immune response Human NKG2D signaling	1.303E-04
	Immune response TSLP signalling	1.445E-04
	Immune response Murine NKG2D signaling	1.936E-04

## Discussion and Conclusion

Identifying cancer subtypes is one of the important components in the personalised medicine framework, as correctly stratifying patients into subtypes will increase the chance to provide the best treatment option. Computational methods have been advanced in the last decade to systematically cluster patients into groups based on their genetic profiles. Especially, SNF is an effective multi-omics data fusion method for stratification of cancer subtypes. Compared with other existing methods, SNF is time efficient and effective in uncovering subtypes with distinct survival patterns. However, similar to other existing methods, SNF is not able to exploit the biological importance of the features in building the model. Therefore, the valuable information from biological networks, such as gene regulatory networks, is not utilised in the procedure of grouping patients into subtypes. However, network information is very important for understanding the mechanisms of cancer development and progression.

In this paper, we have proposed the WSNF method. WSNF is based on SNF, but it makes use of the miRNA-TF-mRNA regulatory network to take the importance of the features into consideration. We applied WSNF to the breast cancer and glioblastoma multiform datasets, and the experimental results have shown that with the assistance of the network information WSNF outperforms the other cancer subtype identification methods that do not use the information. The results suggest that the miRNA-TF-mRNA regulatory network can provide valuable information for clustering cancer subtypes.

The performance of the WSNF method could be improved further. WSNF is based on the public databases of interactions between miRNAs, TFs, and mRNAs. Although the databases are comprehensive, they do not cover all the true interactions. Moreover, the gene regulatory networks may involve other types of molecules such as long non-coding RNAs, and they are not included in the gene regulatory network in this paper. Therefore, a more complete gene regulatory network with multiple types of gene regulators (can be obtained when more data become available) would help improve further the performance of WSNF in identifying significant cancer subtypes.

We have observed that the expression patterns of genes across the identified breast cancer subtypes are very different, suggesting that the expression levels of groups of genes may characterise the cancer subtypes. We have also investigated the expression patterns of genes in the sub-networks around *BCL11A* across different subtypes of breast cancer. The results show that the expression pattern of the genes in Subtype 3, where 73.5% of the patients have triple-negative (ER-, PR- and HER2-), is very different from those in other subtypes. Moreover, functional pathway analysis shows that different pathways involved in different breast cancer subtypes, suggesting that the breast cancer subtypes may be caused by different pathways. These findings are useful for domain experts to design different treatments for different breast cancer subtypes.

In summary, we have developed a method utilising the information of miRNA-TF-mRNA regulatory network to identify cancer subtypes. The method has successfully identified subtypes in breast cancer and glioblastoma multiforme. The results provide strong indicators for further analysis of the mechanisms of the subtypes. We provide all datasets, results and scripts for readers to reproduce and further analyse the results.

## Supporting Information

S1 File TF list(1679). (CSV)

S2 File R scripts for our method. (R)

S3 File Supplementary Materials. (PDF)

S4 File BRCA subtype results with clinical information. (CSV)

S5 File GBM subtype results with clinical information. (CSV)

S6 File The significant differentially expressed genes in the five BRCA subtypes. (CSV)
