# Supplementary material for: Identifying Cancer Subtypes from miRNA-TF-mRNA Regulatory Networks and Expression Data
Source: PLoS One. 2016 Apr 1;11(4):e0152792. doi: 10.1371/journal.pone.0152792 (PMC4818025; doi:10.1371/journal.pone.0152792)
Supplement: S3 File — (PDF) [file pone.0152792.s003.pdf]

Supplementary Materials

1 Supplementary methods

1. **The detailed information of Google PageRank, GeneRank, NCIS (network assisted co-clustering for the identification of cancer subtypes) and Ranking (importance) of features from miRNA-TF-mRNA regulatory network.**

a. Google PageRank

$$\boldsymbol{r} = (1-d) \times (\boldsymbol{I} - d\boldsymbol{E}\boldsymbol{D}^{-1})^{-1} \times \boldsymbol{e}$$

$\boldsymbol{r}$ is an N x 1 vector representing the ranking of each webpage. N is the number of webpages.

$\boldsymbol{d}$ is the damping factor.

$\boldsymbol{I}$ is an N x N identity matrix.

$\boldsymbol{E}$ is a directed N x N matrix representing the links among the webpages.
If there is a page j links to page i, $E_{ij} = 1$; otherwise $E_{ij} = 0$.

$\boldsymbol{D}$ is an N x N diagonal matrix, $D_{jj} = \sum_i E_{ij}$ which means the number of webpages that page j point to.

$\boldsymbol{e}$ is an N x 1 vector with all entries 1.

$$E: \; i \overset{j}{\begin{bmatrix} 0 & 0 & 1 & 1 \\ 1 & 0 & 0 & 1 \\ 0 & 1 & 0 & 1 \\ 1 & 1 & 1 & 0 \end{bmatrix}}$$

$j \rightarrow i$

An example

b. GeneRank

$$\mathbf{r} = (1-d) \times (\boldsymbol{I} - d\boldsymbol{W}\boldsymbol{D}^{-1})^{-1} \times \frac{\boldsymbol{ex}}{max(\boldsymbol{ex})}$$

r is an N x 1 vector represents the weight of each gene. N is the number of genes.

d is the damping factor.

$\boldsymbol{I}$ is an N x N identity matrix.

W is an N x N connectivity symmetric matrix for the gene network.
If there is a correlation between gene i and gene j, $W_{ij} = 1$; otherwise $W_{ij} = 0$.

D is an N x N diagonal matrix, $D_{jj} = \sum_i W_{ij}$.

ex is an N x 1 vector representing the absolute value of the expression difference for each gene. (e.g. variance or median absolute deviation)

c. NCIS

$$\mathrm{r} = (1-\alpha) \times (\boldsymbol{I} - \alpha\boldsymbol{E}\boldsymbol{D}^{-1})^{-1} \times \frac{\boldsymbol{MAD}}{\max(\boldsymbol{MAD})}$$

r is a N x 1 vector representing the weight of each gene. N is the number of genes.

$\boldsymbol{\alpha}$ is the damping factor.

$\boldsymbol{I}$ is an N x N identity matrix.

E is an N x N directed gene-gene regulation matrix.
If gene i regulates gene j, $E_{ij} = 1$; otherwise $E_{ij} = 0$.

D is an N x N diagonal matrix, $D_{jj} = \sum_i E_{ij}$ which means the total number of genes that regulate gene j.

MAD is the N x 1 vector representing median absolute deviation for each gene.

$$E: \; i \overset{j}{\begin{bmatrix} 0 & 0 & 1 & 1 \\ 1 & 0 & 0 & 1 \\ 0 & 1 & 0 & 1 \\ 1 & 1 & 1 & 0 \end{bmatrix}}$$

$i \rightarrow j$

An example

d. Ranking of features from miRNA-TF-mRNA regulatory network

$$\boldsymbol{R} = (1-d) \times (\boldsymbol{I} - d\boldsymbol{E}\boldsymbol{D}^{-1})^{-1} \times \boldsymbol{e}$$

R is an Nx1 vector representing the ranking (importance) of each feature (mRNA, TF or miRNA). N is the number of features (mRNA, TF and miRNA) in the miRNA-TF-mRNA regulatory network.

d is the damping factor.

I is an N x N identity matrix.

E is an NxN directed miRNA-TF-mRNA regulatory network matrix.

If feature i regulates feature j, E_{ij} = 1; otherwise E_{ij} = 0.

D is an NxN diagonal matrix, $D_{jj} = \sum_i E_{ij}$ which means the total number of regulators feature i that regulate feature j.

e is an N x 1 vector with all entries 1.

$$E: i \overset{j}{\begin{bmatrix} 0 & 0 & 1 & 1 \\ 1 & 0 & 0 & 1 \\ 0 & 1 & 0 & 1 \\ 1 & 1 & 1 & 0 \end{bmatrix}}$$

$i \rightarrow j$

An example

2. The Pearson Correlation Coefficient between the GeneRank result and the PageRank result

The dataset is downloaded from the GeneRank Additional files.

(Available at http://www.biomedcentral.com/1471-2105/6/233/additional)

	**The ranking from GeneRank formula** (Integrated with MAD) [Network: 4047x4047]
**Original MAD**	0.0104
**The ranking from PageRank formula** (Without MAD)	**0.9101**

The Pearson Correlation Coefficient between the GeneRank result and the PageRank result is 0.91 which means the MAD information is not effectively integrated in the GeneRank result.

3. The Pearson Correlation Coefficient between NCIS result and PageRank result

The datasets are downloaded from the NCIS Additional files.

(Available at http://bioen-compbio.bioen.illinois.edu/NCIS/)

	**The weight from NCIS formula** (Integrated with MAD **)**	
	BRCA [network: 8726x8726]	GBM [network: 7183x7183]
**Original MAD**	0.0918	0.1
**The ranking form PageRank formula** (without MAD)	**0.9915**	**0.9949**

For the high-dimension BRCA and GBM gene-gene networks, the Pearson Correlation Coefficient between NCIS result and PageRank result are **0.9915** and **0.9949**, respectively. The high correlation implies that the MAD information is not effectively integrated in the NCIS result.

2. Supplementary Figures

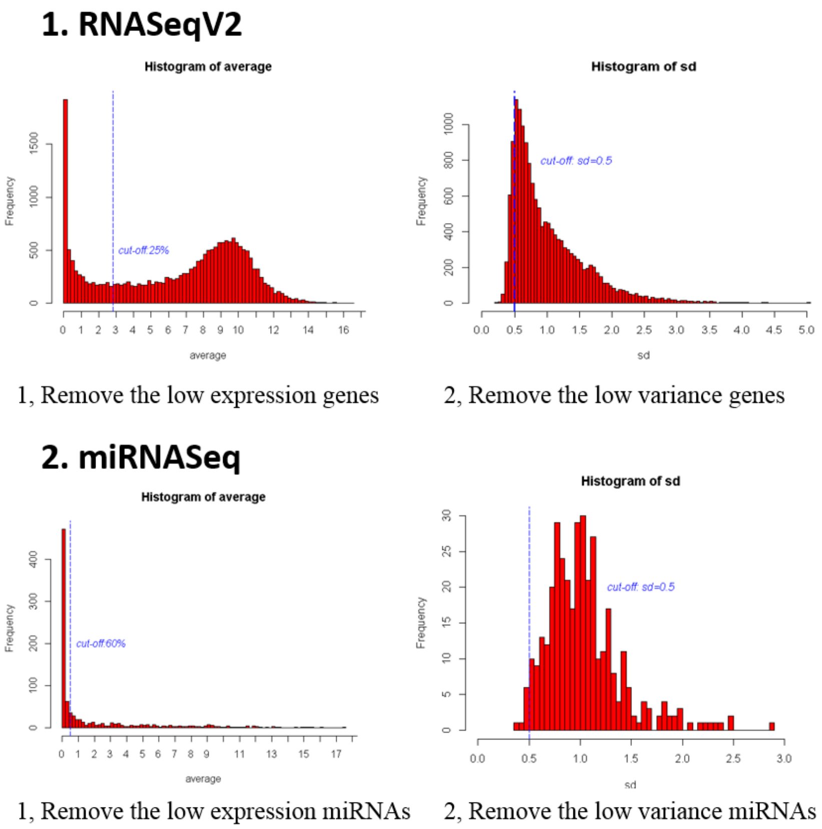

(a)

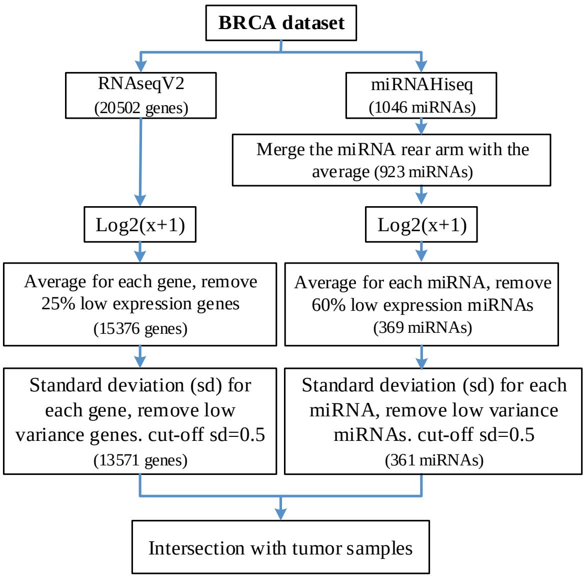

(b)

Fig. S1. The data process of BRCA.

Figure (a) shows the data distribution histogram and the cut-off points.

Figure (b) shows the processing flow of the BRCA RNASeq and miRNASeq datasets.

1. mRNA Expression (microarray)

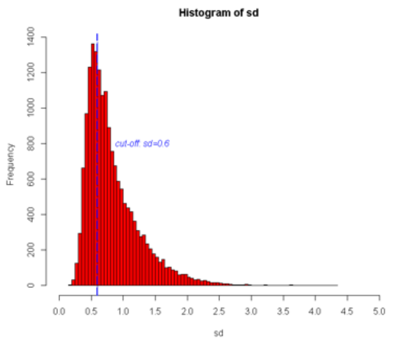

Remove the low variance genes

2. miRNA Expression (Agilent 8x15k)

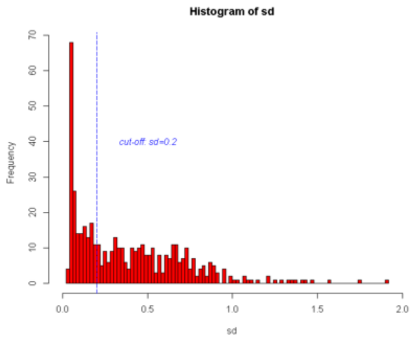

Remove the low variance miRNAs

(a)

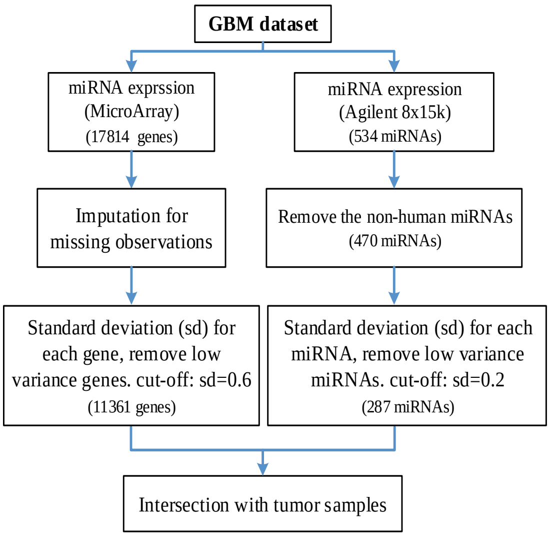

(b)

Figure S2. The data process of GBM.

Figure (a) shows the data distribution histogram and the cut-off points.

Figure (b) shows the processing flow of the GBM mRNA and miRNA datasets.

3. Supplementary Tables

Table S1 The number of the patients and the number of mRNA, TF and miRNA used in the experiments.

Cancer Type	Patients Number	mRNA	TF	miRNA
**BRCA**	587	12,233	1,338	361
**GBM**	276	10,278	1,083	287

Table S2 The interactions used for constructing the miRNA-TF-mRNA regulatory network for the GBM dataset.

	Database	Total interactions	Found interactions
miRNA → TF& miRNA →mRNA	Tarbase v6.0	17,526	10,236
	miRTarBase v4.5	37,423	19,880
	miRecords v4	1,707	1,024
	starBase 2.0	320,709	155,144
	ENCODE [25]	117,193	33,775
TF → miRNA	ENCODE [25]	1,648	546
	Transmir v1.2	649	427
TF → mRNA	ENCODE ChIP-Seq	229,486	82,960
	TRED	7,066	3,921
TF → TF& mRNA → mRNA	Reactome	127,452	50,269
	STRING v10.0	250,843	89,911
